# TP53 Mutation-Specific Dysregulation of Store-Operated Calcium Entry and Apoptotic Sensitivity in Triple-Negative Breast Cancer

**DOI:** 10.3390/cancers17101614

**Published:** 2025-05-10

**Authors:** Kaneez E. Rabab, Paul J. Buchanan, Grace Colley, Anita White, Aisling Murphy, Chloe McCormack, Alex J. Eustace

**Affiliations:** 1 Life Sciences Institute, Dublin City University, D09 NR58 Dublin, Ireland; kaneez.rabab@gmail.com (K.E.R.); grace.colley3@mail.dcu.ie (G.C.); anita.white@dcu.ie (A.W.); aisling.murphy376@mail.dcu.ie (A.M.); chloe.mccormack44@mail.dcu.ie (C.M.); 2 School of Nursing, Psychotherapy and Community Health, Dublin City University, D09 Y8VX Dublin, Ireland; 3 School of Biotechnology, Dublin City University, D09 K20V Dublin, Ireland

**Keywords:** breast cancer, calcium signalling, *TP53* mutations, store-operated Ca^2+^ entry, apoptotic resistance

## Abstract

**Background:** Triple-negative breast cancer (TNBC) is an aggressive subtype lacking estrogen, progesterone, and HER2 receptors, and is associated with poor prognosis and limited targeted therapeutic options. TP53 mutations occur in the majority of TNBC cases, disrupting p53’s role in DNA repair and apoptosis. Beyond gene regulation, p53 also influences calcium signalling through store-operated calcium entry (SOCE), a critical pathway for cell survival and death. However, the impact of different TP53 mutation types on calcium signalling remains unclear. **Methods:** Calcium channel gene expression was analysed using publicly available TNBC datasets. Calcium channel expression and SOCE activity were assessed in TNBC cell lines with different TP53 mutations using quantitative PCR and calcium imaging (Fura-2AM). Cell proliferation was measured using acid phosphatase assays, while apoptosis was evaluated through caspase 3/7 activation using the Incucyte live-cell fluorescent imager. The p53 reactivator COTI-2 was tested for its ability to restore TP53 function and modulate calcium signalling. **Results:** Analysis revealed significant downregulation of CACNA1D in TP53-mutant TNBCs. TNBC cell lines harbouring frameshift and stop TP53 mutations exhibited reduced SOCE, lower CACNA1D expression, and resistance to thapsigargin-induced apoptosis compared to wild-type cells. In contrast, cells with the TP53 R273H missense mutation demonstrated similar calcium signalling and proliferation to TP53 wild-type cels. COTI-2 treatment restored CACNA1D expression and SOCE in frameshift and stop mutant cells, enhancing apoptotic sensitivity. Combined treatment with COTI-2 and thapsigargin resulted in a synergistic increase in apoptosis. **Conclusions:** This study identifies a novel link between TP53 mutation type and calcium signalling in TNBC. Reactivating mutant p53 with COTI-2 restores calcium-mediated apoptosis, supporting combination strategies targeting both TP53 dysfunction and calcium signalling.

## Simple Summary

Triple-negative breast cancer (TNBC) is an aggressive and hard-to-treat form of breast cancer that has limited treatment options. In TNBC, changes in DNA (mutations) associated with the cancer, frequently occur in the TP53 gene. The TP53 gene normally helps cells repair damage or self-destruct when they are not functioning properly; however when mutated, TP53 cannot function properly. In this study, we found that different types of TP53 mutations, affect how cancer cells manage calcium—an important signal that controls how cells grow and die. Some of these mutations made the cancer cells less likely to die under stress, which may explain why they can resist certain treatments. To address this problem, we tested a TP53 reactivator that helps restore the normal functions of TP53. This TP53 reactivator increased calcium levels in the cancer cells and made them more responsive to treatment. Our findings help explain why some cancers are more aggressive and suggest a new approach to treatment by targeting both the mutant TP53 and the disrupted calcium signals. This could lead to better therapies and improved outcomes for people with TNBC.

## 1. Introduction

Breast cancer (BC) remains the most commonly diagnosed cancer among women worldwide, accounting for approximately 23.8% of all new cancer cases in females in 2022 [1]. It is a highly heterogenous and genetically complex disease, encompassing various molecular subtypes defined by distinct expression patterns of cell surface receptors [2]. Triple-negative breast cancer (TNBC), also known as basal-like BC, constitutes 10–15% of BC cases. It is characterised by the absence of estrogen (ER), progesterone (PR), and human epidermal growth factor receptor 2 (HER2) expression. As a result, this subtype does not respond to receptor-targeted therapies used for ER+ and HER2+ BC [2]. TNBC’s aggressive nature and limited availability of effective treatments contribute to its poorer survival rates [3]. Consequently, non-specific chemotherapy remains a key standard of care treatment, but less than 30% of patients achieve a complete response [3]. Moreover, TNBC exhibits the highest recurrence and mortality rates among BC subtypes [3]. Therefore, there is an urgent need to develop new therapeutic strategies to improve TNBC patient outcomes.

Tumour protein 53 (*TP53*) is a tumour suppressor gene (TSG) that encodes the p53 protein, known to play a major role in controlling cell division. Activated in response to cellular stressors, including DNA damage, p53 induces cell-cycle arrest, DNA repair, and apoptosis [4,5]. *TP53* is the most mutated gene in human cancer, with a frequency of 40–50%. In TNBC, *TP53* mutations are highly prevalent (~60–80%), occurring more frequently than in other BC subtypes [6]. However, these mutations are diverse in nature, with missense (~70%), stop (~10%), and frameshift (FS, ~10%) variants being the most common [7]. This heterogeneity complicates our understanding of their individual impact, as different mutations can lead to loss of wild-type p53 tumour suppressor function and/or acquisition of gain-of-function oncogenic properties [8]. Despite this, research has shown that certain *TP53* mutants have been linked with enhanced invasion, migration, tumour survival, and resistance to apoptosis, which collectively contribute to a poorer prognosis and chemoresistance [9,10]. As a result, significant efforts have focused on targeting the mutant form of the resultant protein, p53, as a therapeutic strategy [8]. This has led to the development of novel p53 reactivators, which can restore p53 wild-type conformation and downregulate mutant expression [11,12]. *TP53* restoration results in anti-tumorigenic effects such as inhibition of cancer proliferation and apoptosis induction [13,14]. Despite these advances, clinical trials have reported variable patient responses [15], highlighting the need for a more comprehensive understanding of the mechanisms underlying *TP53* mutations to predict treatment outcomes and refine therapeutic strategies.

A key function of p53 is its ability to induce apoptosis through a well-established transcriptionally-dependent pathway, upregulating pro-apoptotic genes such as PUMA (p53 upregulated modulator of apoptosis) and NOXA (a BH3-only protein that promotes apoptosis) [16]. This process activates caspases and promotes cytochrome C release via dimerisation of BAX and BAK on the mitochondria [17]. Emerging research has demonstrated that p53 can also induce apoptosis via a transcriptionally-independent mechanism, notably through the modulation of intracellular calcium (Ca^2+^) [18]. In non-excitable cells, Ca^2+^ homeostasis is primarily regulated by store-operated current (SOC), which mediates Ca^2+^ release from the endoplasmic reticulum (ER) and facilitates Ca^2+^ entry through store-operated Ca^2^ entry (SOCE) across the plasma membrane (PM). This mechanism is integral to key cellular processes, including proliferation, survival, and apoptosis [19]. Aberrations in SOC activity have been linked to cancer progression, including BC [20,21,22], where they support hallmarks of malignancy such as proliferation, migration, and apoptotic resistance [23,24,25].

Notably, p53 has been shown to interact with SOC [18,26], facilitating Ca^2+^ loading into the endoplasmic reticulum (ER) via sarcoplasmic/ER Ca^2+^-ATPase (SERCA) [27] and promoting SOCE by regulating Ca^2+^ channels at the PM, such as transient receptor potential channel (TRPC6) [28]. Furthermore, by mediating Ca^2+^ transfer between the ER and mitochondria, p53 triggers apoptosis through the activation of caspase-3 and the cleavage of PARP [29]. However, studies have highlighted that disruption to p53 function or associated Ca^2+^ homeostasis can promote apoptotic resistance and reduced chemotherapy sensitivity [27,29]. Further research is required to understand how different *TP53* mutations influence this pathway and to assess its therapeutic potential.

This study aimed to elucidate the impact of *TP53* mutations on SOC activity and its contribution to apoptotic resistance in TNBC cells. Our findings demonstrate that *TP53* mutations differentially impact Ca^2+^ channel expression and SOC activity. Specifically, certain FS or stop *TP53* mutations contribute to apoptotic resistance by downregulating SOC activity. Notably, this effect can be restored through the use of p53 reactivators. This first-of-its-kind study provides critical insights into how *TP53* mutation type can influence apoptotic sensitivity through Ca^2+^ modulation. Furthermore, our data suggest that targeting SOC channels in combination with p53 reactivation could yield synergistic therapeutic effects, offering a novel strategy for overcoming TNBC treatment resistance.

## 2. Materials and Methods

### 2.1. Bioinformatics

Differential expression analyses (DEAs) were carried out using Cancer Cell Line Encyclopaedia (CCLE) BC cell line microarray data and The Cancer Genome Atlas (TCGA) BC patient sample RNA-sequencing data using Bioconductor software packages (version 3.16, BiocManager 1.30.23) in RStudio (Version 4.2.2, release date 2022-10-31). Differentially expressed genes (DEGs) with log_2_ fold change (lfc) ≥ 2 and Benjamini-Hochberg adjusted (BH-adj). *p*-value ≤ 0.01 were identified in all BC subtypes, *TP53* MUT vs. WT and TNBC/Basal *TP53* MUT vs. all BC subtype WT. Volcano plots were generated to illustrate the DEGs. The DEGs were cross-analyzed using Venn diagrams to identify overlapping targets between datasets. Ca^2+^ genes were identified from the overlapping targets.

### 2.2. Cell Culture

This research employed several TNBC cell lines, grouped based on their *TP53* mutation status, with CAL-51 (ATCC) representing *TP53* wild-type. HDQ-P1 (DMSZ) (R213*) and MDA-MB-157 (ATCC) (A88fs) displayed rarer but more deleterious stop (nonsense) and frameshift mutations, respectively [30,31]. While MDA-MB-468 (R273H) and MFM223 (K132R) (both ATCC) harbour more common single nucleotide variant (SNV) missense mutations [32]. Each cell line was cultured in the outlined specific media; CAL-51-DMEM (Sigma-Aldrich, Merck KGaA, Dramstadt, Germany) 10% Fetal Bovine Serum (FBS; Gibco); HDQP1-DMEM 10% FBS and 2 mM L-glutamine (Gibco, Thermo Fisher Scientific, Waltham, MA, USA); MDA-MB-157-L15 (Corning®, Corning Inc., Corning, NY, USA) 10% FBS; MDA-MB-468-RPMI (Sigma-Aldrich) 10% FBS; MFM223-MEM (Sigma Aldrich) 10% FBS. Grown in T75 flasks at 37 °C in a humidified incubator containing 5% carbon dioxide. Cell lines were treated with sarco/endoplasmic reticulum Ca^2+^ ATPase (SERCA) inhibitor/ER stressor, thapsigargin (TG) (T9033, Sigma-Aldrich), prepared to 10 mM in DMSO. In addition, COTI-2, a third-generation thiosemicarbazone p53 reactivator (Selleckchem, Houston, TX, USA; Cat. No. S8580), was prepared at a concentration of 5 mM in DMSO. Restoring p53 function by binding to misfolded mutant p53 and inducing a conformational change that reactivates its tumour suppressor activity [33,34]. Due to its greater potency compared to other p53 reactivators, such as APR-246, and its higher specificity relative to broader-acting compounds such as α-mangostin, COTI-2 was selected as the most suitable agent for this investigation [35,36].

### 2.3. CACNA1D siRNA Knockdown

siRNA knockdown was performed on CAL-51 cell line to induce knockdown of *CACNA1D* gene, which codes for the protein Cav1.3. Cells were seeded into 6-well plates at 4 × 10^5^ cells per well, achieving 80% confluency following overnight incubation. Followed by treatment with either siRNA targeting gene *CACNA1D* (Si*CACNA1D*, Silencer Select, Thermo Fisher Scientific, Waltham, MA, USA; Cat. No.; 4392420) or siRNA non-targeting negative control (SiNeg, Silencer Select, Thermo Fisher Scientific, Waltham, MA, USA; Cat. No. 4390843). Prepared by adding 30 pmol of siRNA to Opti-MEM media (Sigma, Merck KGaA, Dramstadt, Germany) and separately 9 µL of Lipofectamine 3000 (Invitrogen, Thermo Fisher Scientific, Waltham, MA, USA) to Opti-MEM media. After 5 min, both tubes were mixed and left at room temperature (RT) to incubate for a further 20 min. Afterwards, culture media were removed from wells, and 250 µL of the transfection mix was added to the appropriate wells at a final concentration of 25 pmol of siRNA and 7.5 µL of Lipofectamine 3000, before making up the final volume per well to 2 mls with opti-MEM media. The plate was incubated at 37 °C, 5% CO_2,_ for a period of 48 h ahead of qPCR or SOCE assays.

### 2.4. Real-Time PCR

Gene expression was assessed in the TNBC cell lines with or without 24 h COTI (100 nM) treatment. A High Pure RNA Isolation Kit (Roche, F. Hoffmann-La Roche Ltd., Basel, Switzerland) was used to extract RNA as per the manufacturer’s guidelines. Total RNA was quantified using a nanodrop, with purity determined through the A_260_/A_280_ ratio. Lyophilised primers (Sigma Aldrich, Merck KGaA, Dramstadt, Germany) were designed based on an exon-spanning sequence. Gene expression was measured using a One-step Luna qPCR kit (New England Biolabs, Ipswich, MA, USA) with the reaction mixture according to the kit instructions, including 10 μM primers (Sigma Aldrich, Merck KGaA, Dramstadt, Germany) (Supplementary Table S1) and 400 ng RNA. Loading three biological replicates for each cell line on a 96-well PCR plate, they were analysed using an Applied Biosystems QuantStudio 3 (Thermo Fischer Scientific, Waltham, MA, USA) instrument. The fold change in gene expression was calculated using the 2^−ΔΔCT^ method with β-Actin as the housekeeping gene and CAL-51 (TNBC WT cell line) as the control group for baseline gene expression. The gene expression of the COTI-2-treated cell lines was normalised to the expression of the untreated cell lines.

### 2.5. Calcium Measurement

Relative changes in cytosolic calcium (Ca_c_^2+^) were evaluated as previously described [37]. In brief, TNBC cells were grown in black 96-well plates before loading for 1 h in the dark at 37 °C with ratiometric Ca^2+^ dye Fura-2-AM (2 mM, Abcam, Abcam Ltd., Cambridge, UK) diluted in DMEM 10% FCS. Thereafter, the cells were washed with DMEM 10% FCS and incubated for 10 min, prior to being resuspended in 100 μL of Ca^2+^ free physiological saline solution (PSS, in mM NaCl 140, MgCl_2_ 1, KCl 4, D-glucose 11.1 and HEPES 10, EGTA 1, adjusted to pH 7.4 with NaOH). A VICTOR multilabel plate reader was used to measure fluorescence at 510 nM after excitation at 340 and 380 nM to record SOC over 12 min. Basal cytosolic Ca^2+^ was measured for 100 s, followed by endoplasmic reticulum depletion induced by 5 µM TG and read for 400 s, before application of 2 mM CaCl_2_ to record store-operated Ca^2+^ entry (SOCE) for a further 200 s. Changes in basal Ca^2+^ were calculated by taking an average of the first 100 s. Changes in Ca_c_^2+^ resulting from ER store release or Ca^2+^ entry following application of TG or CaCl_2_, respectively, were calculated by taking the average baseline away from the maximal peak value.

### 2.6. Proliferation Assay

MDA-MB-157, CAL-51 and HDQ-P1 were treated with or without TG (100 nM) for 5 days to promote ER stress-induced cell death. Proliferation was determined with the acid phosphatase assay as described previously [38]. All media were removed, and the wells were washed once with PBS. Paranitrophenol phosphate substrate (0.263 g of PNP in 100 mL sodium acetate buffer) was added to each well and incubated at 37 °C for 2 h. 50 μL of 1 M NaOH was added, and the absorbance was read at 405 nM (reference—620 nM). Inhibition of proliferation analysis was calculated as a percentage of untreated controls, with the Chou–Talalay equation via CalcuSyn ™ Version 2.0 used to determine the effective dose of the drug that inhibits 50% of growth (IC_50_).

### 2.7. Apoptosis Assay

IncuCyte’s live-cell imaging system was used to determine apoptosis induction. On a single 96-well plate, 3 × 10^3^ cells/well for each TNBC cell line were seeded before treatment with TG and COTI-2, at the indicated drug concentrations. Control wells were treated with media or DMSO, or were untreated. All assays were repeated in triplicate. Caspase 3/7 green dye (Sartorius AG, Gottingen, Germany; Cat. No. 4440) was loaded into each well as recommended, before the plate was incubated in the IncuCyte at 37 °C in 5% CO_2_. Apoptosis was detected when activated caspase 3/7 cleaved the inert dye, releasing the fluorescent dye, which was measured at 530 nM. IncuCyte 2022 A software was used for plate setup and continuous data acquisition of fluorescent signals at 4-h intervals over a 5-day period. The software automatically presented real-time kinetic changes in cell number and caspase 3/7 activation. Graphs and statistical analyses were performed using GraphPad Prism 7.

### 2.8. Statistical Analysis

The graphs were all prepared using the Prism software (GraphPad software, Inc., SanDiego, CA, USA). Except otherwise stated, results are reported as means +/− standard errors of the mean. A non-parametric test was used to assess non-normal sample distributions and N < 10. A Mann-Whitney test or a Wilcoxon test was used for non-parametric tests between two groups, and a Kruskal-Wallis test with Dunn’s multiple comparison test (MCT) was used for non-parametric tests between multiple groups. Statistical tests used are indicated in each figure legend, with significance considered as * *p* < 0.05, ** *p* < 0.01, *** *p* < 0.001 and **** *p* < 0.0001. Unless otherwise noted, all results are generated from at least three independent biological experiments denoted by N, with a total number of individual repeats in each experiment denoted by n.

## 3. Results

### 3.1. TP53 Mutations Associated with Differential Ca^2+^ Channel Gene Expression in TNBC

**Figure 1:**
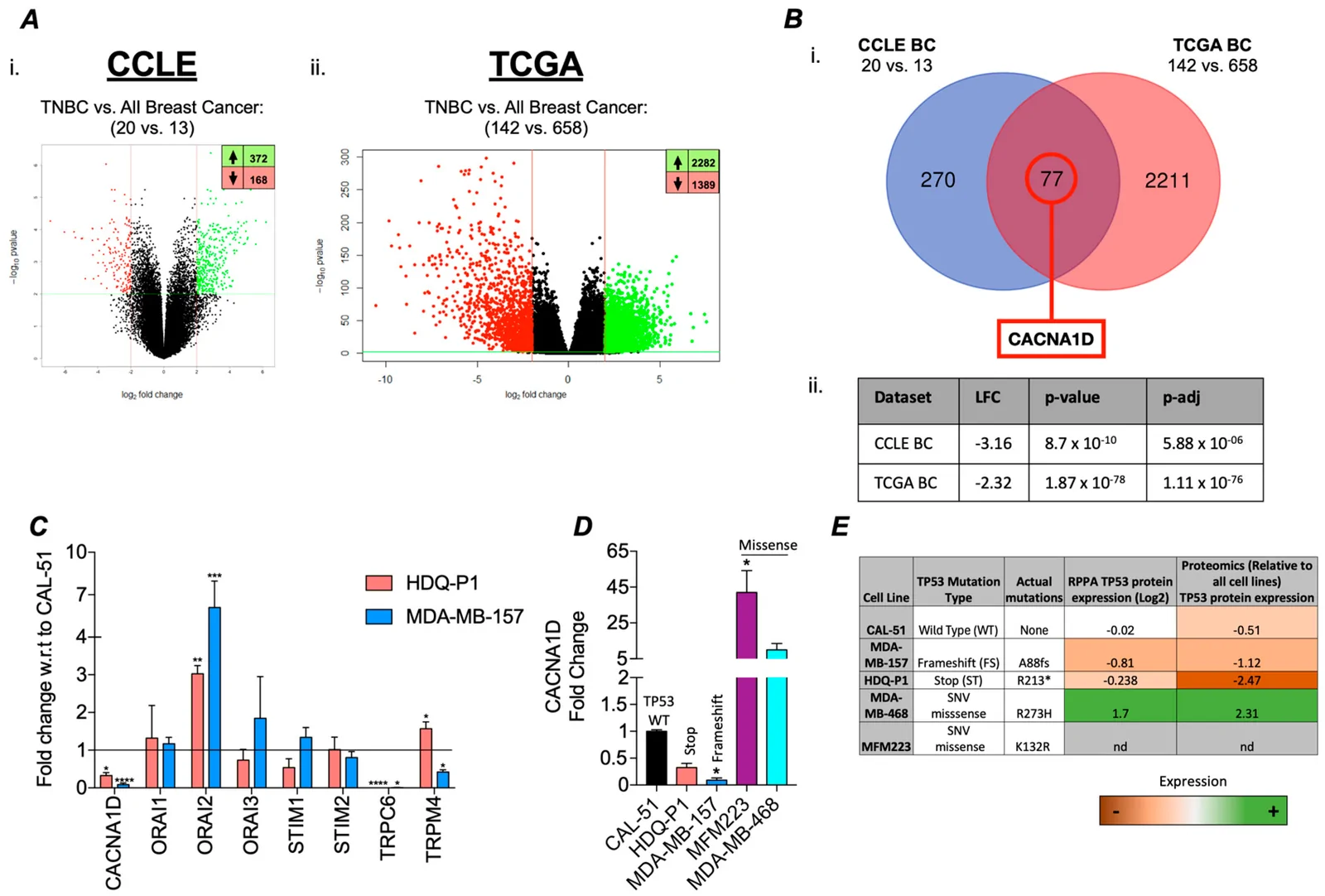
Differential calcium channel gene expression in TNBC TP53 mutant patient samples and cell line versus TP53 wildtype. (**A**) Differential expression analyses (DEAs) carried out using CCLE breast cancer cell line microarray data and TCGA patient sample RNA-sequencing data. (i) The CCLE volcano plot illustrates the DEGs (Dots) when comparing TNBC TP53 MUT (n = 20) vs. all BC subtype WT (n = 13) with 372 significantly upregulated (lfc ≥ 2, BH-adj. *p*-value ≤ 0.01) and 168 significantly downregulated (lfc ≤ −2, BH-adj. *p*-value ≤ 0.01) genes. and (ii) the TCGA volcano plot illustrates the DEGs when comparing TNBC TP53 MUT (n = 142) vs. all BC subtypes WT (n = 658) samples with 2282 significantly upregulated and 1389 significantly downregulated genes. (**B**) (i) Differentially expressed genes from the CCLE BC and TCGA BC analyses were cross-analysed using a Venn diagram to identify overlapping targets between datasets. (ii) Calcium channel genes were searched for in the overlapping targets, with only *CACNA1D* identified as significantly differentially expressed, with details displayed in a table with relevant lfc, *p*-value, and p-adj (BH-adjusted). (**C**) Bar graph displaying relative fold change of calcium channel gene expression in TP53 mutant TNBC cell lines HDQ-P1 (red) and MDA-MB-157 (blue) with respect to (w.r.t.) to TP53 wild-type TNBC cell line CAL-51 using qRT-PCR. (**D**) shows the gene expression of *CACNA1D* in expanded panel of TNBC cell lines include missense mutants, MFM223 and MDA-MB-468 (n = 4). Kruskal-Wallis with Dunn’s multiple comparisons was used to determine significance. (**E**) Table outlining the cell lines used in this study, including their corresponding TP53 mutation status and type, as well as associated p53 protein expression levels retrieved from the CCLE.

To determine the impact of *TP53* mutations on gene expression, we performed a differential gene expression analysis (DGEA) between *TP53* mutant TNBC to wild-type *TP53* BCs (All subtypes). Our analysis revealed the significant dysregulation of 372 upregulated and 168 downregulated genes in the CCLE (cell line samples) dataset, and 2282 upregulated and 1389 downregulated genes in the TCGA (patient samples) dataset (Figure 1(Ai,Aii). From these datasets, we identified 77 genes that were commonly dysregulated in both datasets with a significant log_2_ fold change (log_2_FC) ≥ 2 (Figure 1(Bi)). Among these, *CACNA1D*, an L-type voltage-gated Ca^2+^ channel, was the only Ca^2+^ channel gene to exhibit significant differential expression, with downregulated expression in *TP53* mutant samples in both the CCLE (log_2_FC = −3.16) and TCGA (log_2_FC = −2.32) datasets (Figure 1(Bii)). In the TCGA dataset, *CACNA1D* was also significantly downregulated in TP53-mutant samples across all breast cancers (lfc = −1.30; adj. *p* = 1.43 × 10^−41^), but the effect was less, suggesting a subtype-specific association between *CACNA1D* loss and TP53 mutation in TNBC. Furthermore, our analysis demonstrated in the TCGA database that in BC samples, reduced *CACNA1D* expression was associated with a significant reduction in disease-free survival (Supplementary Figure S1A,B) (HR: 1.604 (95% CI: 1.109–2.320), *p* < 0.0127, q < 0.0254). This effect was also evident in TNBC but failed to reach significance (HR: 1.402 (95% CI: 0.556–3.537)). Overall, this highlights *CACNA1D* as a target of clinical importance.

**Figure 2:**
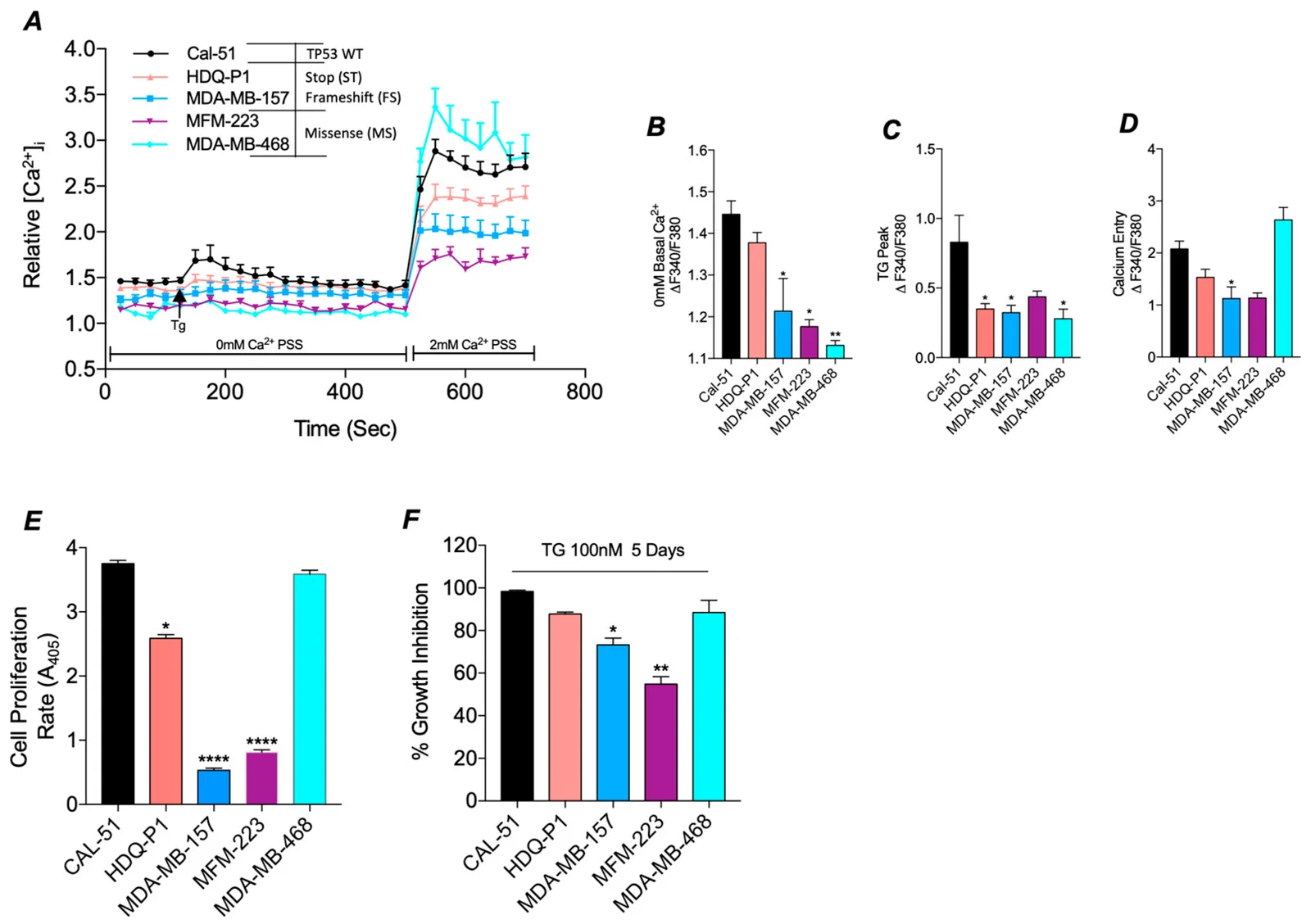
SOC and tumour biology is altered in TP53 mutant TNBC cell lines compared to WT. (**A**) Traces of SOC were measured in TNBC cell lines, either TP53 WT CAL-51 (black, n = 7) or mutant HDQ-P1 (red, n = 8), MDA-MB-157 (blue, n = 7), MFM-223 (Purple, n = 4) and MDA-MB-468 (Cyan, n = 4). ER Store release was induced by 4 μM TG and SOCE by the addition of 2 mM Ca^2+^. Trace analysis measured (**B**) basal calcium in 0 mM Ca^2+^, (**C**) TG peak and (**D**) SOCE peak between cell lines. (**E**) Presents the rate of proliferation read-out from the acid-phosphatase assay in TP53 mutant TNBC cell lines compared to the TP53 wild-type TNBC cell line (n16, n = 4). (**F**) Displays percentage growth inhibition after treatment with ER stressor thapsigargin (100nM, TG) for 5 days compared to DMSO control as measured by acid-phosphatase assay. For all above Kruskal-Wallis with Dunn’s multiple comparisons was used to determine the significance.

To validate this bioinformatic analysis, we measured the expression of a panel of Ca^2+^ channels linked to SOC in *TP53* mutant cell lines HDQ-P1 and MDA-MB-157. Compared to the wild-type (WT) *TP53* CAL-51 cell line, we observed a significant reduction in *CACNA1D*, *TRPC6*, and *TRPM4* expression, alongside an upregulation of *ORAI2* (Figure 1C). Given that *CACNA1D* produced the largest FC and considering the impact of different TP53 mutant types on survival outcomes in various cancers, including TNBC [39,40], we measured its expression in a broader panel of *TP53* mutant TNBC cell lines. Identifying differential expression based on *TP53* mutation type (Figure 1D). Cells harbouring more deleterious mutations, such as stop and frameshift mutations, including HDQ-P1 and MDA-MB-157, displayed a significant 3-fold and 11-fold decrease in *CACNA1D* expression, respectively, compared to CAL-51 (*p* < 0.05; Figure 2D). In contrast, cells with single-nucleotide variant (SNV) missense mutations, such as MDA-MB-468 and MFM-223, exhibited a 42-fold and 10-fold increase in *CACNA1D* expression relative to CAL-51 (*p* < 0.05). Changes in *CACNA1D* expression correlated with *TP53* expression as outlined in Figure 1E. HDQ-P1 and MDA-MB-157, which have FS or Stop mutations that had low *CACNA1D,* also displayed lower levels of P53 expression. Alternatively, MDA-MB-468, which harbours a missense mutation and exhibited increased *CACNA1D* expression, demonstrated enhanced p53 expression. Collectively, these results indicated that *TP53* mutation type influences Ca^2+^ channel expression in TNBC cells.

### 3.2. Mutant TP53 Impacts SOC and Proliferation in TNBC

To determine if the differential expression of *CACNA1D* in *TP53* mutant TNBC cells had a functional impact, we measured SOC activity (Figure 2A). Measurements of basal cytosolic calcium (Ca_c_^2+^) levels, relative to CAL-51, revealed a reduction in levels across all *TP53* mutant cell lines, with a significant decrease observed in MDA-MB-157 (*p* < 0.05), MFM-223 (*p* < 0.05), and MDA-MB-468 (*p* < 0.01) (Figure 2B). In addition, all *TP53* mutants failed to induce ER store release following TG treatment compared to CAL-51; with HDQ-P1, MDA-MB-157 and MDA-MB-468 displaying significant reductions in ER store release (*p* < 0.05, Figure 2C). Subsequent analysis of SOCE demonstrated a reduction in nearly all *TP53* mutant cell lines relative to CAL-51, reaching significance in MDA-MB-157 (*p* < 0.05). In contrast, the missense mutant line, MDA-MB-468, exhibited a significant increase compared to Cal-51 (Figure 2D) (Reaching significance with Mann-Whitney vs. Cal-51 alone, *p* < 0.01).

Since altered SOC activity is known to influence proliferation and apoptosis, we next assessed the differences in proliferation rates and apoptotic sensitivity between *TP53* mutant and WT cells [41]. Proliferation assays revealed significantly reduced proliferative rates in *TP53* mutant cell lines with HDQ-P1 (*p* < 0.05), MDA-MB-157 (*p* < 0.0001) and MFM-223 (*p* < 0.0001) compared to *TP53* WT CAL-51 cells (Figure 2E), correlating with a reduction in SOC. However, the rate of proliferation in the *TP53* mutant line, MDA-MB-468, which displays higher levels of SOCE, was comparable to WT CAL-51 (Figure 2D).

We next sought to estimate anti-proliferative sensitivity by comparing growth inhibition in cells treated with ER stressor TG relative to DMSO control. We identified a reduction in growth inhibition in *TP53* mutant lines that also had a reduction in SOC, reaching significance in MDA-MB-157 (*p* < 0.05) and MFM-223 (*p* < 0.01) compared to *TP53* WT (Figure 2F). In contrast, CAL-51 and MDA-MB-468, both of which exhibited higher SOCE, displayed greater anti-proliferative sensitivity, with cell death rates of 98% and 88%, respectively (*p* < 0.0001; Figure 2F). These results suggest that mutant *TP53* promotes altered Ca^2+^ channel expression and SOC, which in turn can impact proliferation rates.

### 3.3. Mutant TP53 Modulates SOCE Through CACNA1D

**Figure 3:**
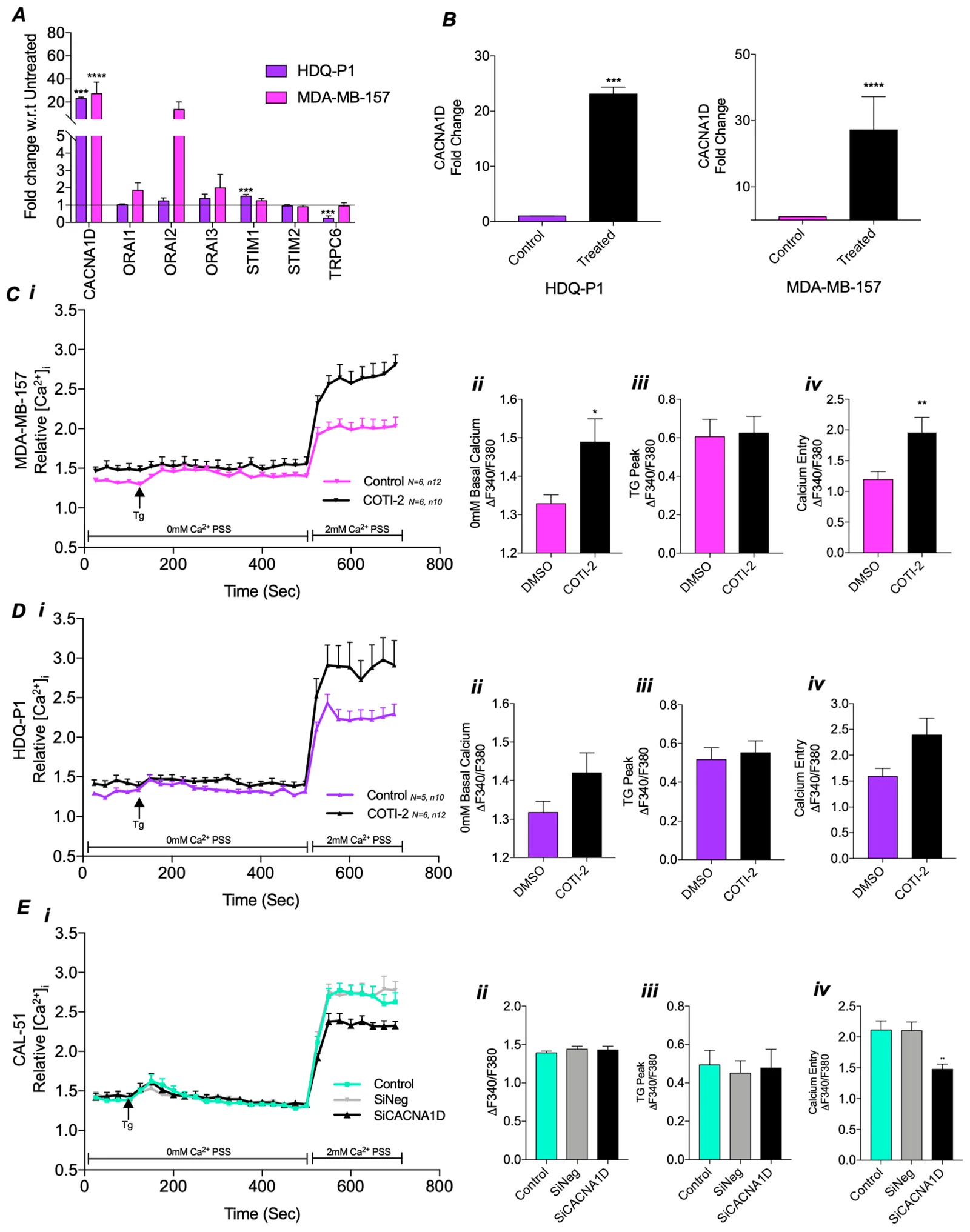
Impact of *CACNA1D* manipulation through COTI-2 treatment or siRNA on calcium channel expression and SOC in TP53 mutant TNBC cell lines. (**A**) Effect of COTI-2 treatment on relative fold change gene expression of calcium channels using qRT-PCR in HDQ-P1 cells (purple) and MDA-MB-157 cells (pink) compared to untreated control. (**B**) Individual graphs of *CANCA1D* relative fold change gene expression in COTI-2 treated TP53 mutant cells vs untreated. SOC measured by Fura-2AM ratiometeric in (**C**) HDQ-P1 or (**D**) MDA-MB-157 as displayed as a trace over time(s) (i) compared to DMSO control, with individual analysis displaying (ii) average baseline 0 mM calcium, (iii) max TG peak and (iv) SOCE. Mann-Whitney was used for all above comparing two groups. (**E**) SOC trace for CAL-51 following (Ei) *CACNA1D* siRNA compared to siNegative or untreated control with individual analysis displaying (ii) average baseline 0 mM calcium, (iii) max TG peak and (iv) SOCE. Kruskal-Wallis with Dunn’s multiple comparisons was used for analysis in (**E**).

To investigate if altered Ca^2+^ channel expression or SOCE dysregulation is associated with mutations in *TP53*, COTI-2, a p53 reactivator, was used. qPCR analysis demonstrated that the expression of Ca^2+^ channels associated with SOC was altered following COTI-2 treatment (Figure 3A). In particular, COTI-2 significantly increased *CACNA1D* expression by 22-fold in HDQ-P1 (*p* = 0.001) and 28-fold in MDA-MB-157 (*p* < 0.0001) compared to the untreated DMSO control (Figure 3B). However, in missense *TP53* mutant lines, MFM-223 and MDA-MB-468, COTI-2 did not affect *CANCA1D* or other SOC channel expression (Figure S2A).

To evaluate the functional impact of the COTI-2-induced changes on Ca^2+^ channel expression, we measured SOC activity Figure 3C(i). In *TP53* mutant MDA-MB-157 cells, COTI-2 treatment led to a significant increase in basal cytosolic Ca^2+^ levels (*p* < 0.05, Figure 3C(ii)) and SOCE (*p* < 0.01, Figure 3C(iv)), restoring levels similar to those observed in CAL-51 WT cells (Figure 2A). However, COTI-2 did not affect TG-induced ER store release (Figure 3C(iii)). Repeating the experiment with HDQ-P1 cells revealed similar increases in basal cytosolic Ca^2+^ levels (Figure 3D(ii)) and SOCE (Figure 3D(iv)), comparable to CAL-51, although these changes were not statistically significant. COTI-2 again failed to impact ER store release (Figure 3D(iii)). In contrast, no differences in SOC were observed between COTI-2-treated and DMSO control groups in missense mutant, MFM-223 and MDA-MB-468 cells (Figure S2B,C). 

These results indicate a potential link between *CACNA1D* expression and SOC. To confirm this relationship, we used siRNA to knock down *CACNA1D* in *TP53* WT CAL-51 cells, which have higher levels of *CACNA1D* expression and SOC. Knockdown was confirmed via qPCR, showing a significant 59% reduction in *CACNA1D* expression (*p* = 0.0286, Figure S3). SOC measurements in this knockdown model revealed a significant reduction in SOCE (*p* < 0.001, Figure 3E(iv)) compared to the siNegative control, reaching levels similar to those in stop and FS *TP53* mutant cells (Figure 2A–D). However, no differences were observed in basal cytosolic Ca^2+^ or ER store release (Figure 3E(ii,iii)). These findings highlight that *TP53* influences SOCE by regulating *CACNA1D* expression, with effects dependent on *TP53* status.

### 3.4. COTI-2 Increases Apoptosis and Sensitivity to Treatment in Both TP53 Wild-Type and Mutant TNBC

**Figure 4:**
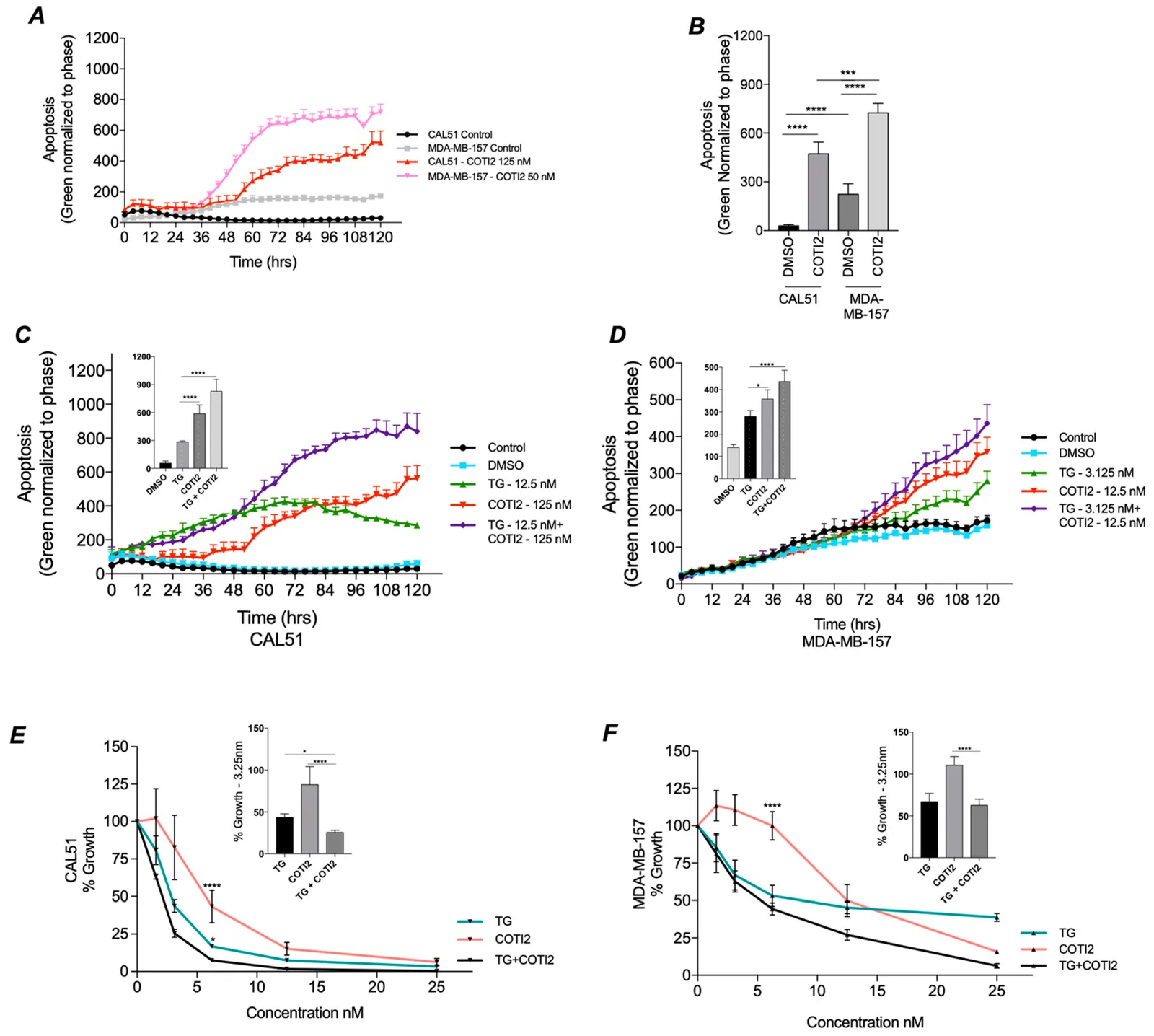
Impact of COTI-2 and TG treatment on Apoptosis. TP53 wildtype CAL-51 and TP53 mutant MDA-MB-157 cells were analyzed for apoptosis following TG and COTI-2 treatment along with DMSO and untreated controls in duplicates. Using the caspase 3/7 dye, apoptosis was recorded every 4 h over a period of 5 days. (**A**) Shows a trace of apoptotic induction in CAL-51 and MDA-MB-157 in the presence and absence of COTI-2. (**B**). Responses to (An) at 120 h where graphed and analysed by Mann-Whitney. Apoptosis traces in CAL51 (**C**) and MDA-MB-157 (**D**) cells under control (black), DMSO (blue), thapsigargin (TG; green), COTI-2 (red), and combined TG+COTI-2 (purple) treatments are shown. Analysed by Two-way ANOVA Post hoc Tukey. Cell proliferation rates after treatment with TG (green), COTI-2 (red) and TG+COTI-2 (black) measured by acid phosphatase in (**E**) TP53 WT CAL-51 and (**F**) T53 mutant MDA-MB-157, with bar graph analysis at 3.125nm. Analysed by One-way ANOVA with post hoc.

Our previous results demonstrated that deleterious *TP53* mutants reduced SOC, leading to decreased proliferation. Since COTI-2 treatment restored *CACNA1D* expression and SOCE in the *TP53* mutant MDA-MB-157 cell line (Figure 3), we investigated whether it could reverse apoptotic resistance. Measuring caspase 3/7 levels, it was observed that COTI-2 alone resulted in significant apoptotic induction in both CAL-51 (*p* < 0.0001) and MDA-MB-157 (*p* < 0.0001) at 120 h, with the *TP53* mutant line displaying greater apoptotic sensitivity (*p* < 0.001, Figure 4A,B). This was corroborated by COTI-2 dose response curves, where MDA-MB-157 had a lower IC_50_ (16.3 nM) compared to CAL-51 (74.3 nM) (Figure S2B). Additionally, basal apoptosis levels were 7.6-fold higher in MDA-MB-157 than CAL-51 (*p* < 0.0001, Figure 4B). These results are potentially attributed to a greater restoration of *TP53* expression/activity, which is lost in *TP53* mutant cells.

Given the reduced anti-proliferative sensitivity of *TP53* mutant lines to ER stressor TG (Figure 2E,F), we assessed whether combining TG with COTI-2 could restore apoptotic sensitivity (Figure 4C,D). Using *TP53* WT CAL-51 as a control, TG and COTI-2 were tested at their IC_50_ concentrations for both cell lines (Figure S4A,B). In CAL-51 (Figure 4C), TG and COTI-2 each significantly increased apoptosis at 120 h versus DMSO control. In CAL-51 cells, the combination of TG and COTI-2, however, produced a synergistic response exceeding TG (*p* < 0.0001) or COTI-2 (*p* < 0.0001) alone. In *TP53* Mutant MDA-MB-157 cells (Figure 4D), both TG and COTI-2 alone significantly increased apoptosis at 120 h versus DMSO control. Their combination again produced a synergistic increase in apoptosis compared to COTI-2 alone (*p* < 0.017) or TG alone (*p* < 0.0001). These results suggest that restoring SOC Ca^2+^ with COTI-2 enhances apoptosis, highlighting its potential for improving treatment sensitivity.

The combined impact of COTI-2 and TG on proliferation was also assessed. In CAL-51 combined TG and COTI-2 treatment produced a synergistic 75% reduction in proliferation (Figure 4E), exceeding the effects of TG alone (53%, *p* < 0.05) or COTI-2 alone (18%, *p* < 0.0001). In the *TP53* mutant MDA-MB-157 line (Figure 4F), combined treatment with COTI-2 and TG produced an enhanced anti-proliferative effect, although this did not reach significance. These results corroborate our previous observations (Figure 2F) that MDA-MB-157 are less sensitive to TG treatment but exhibit synergistic responses when treated with TG and COTI-2.

## 4. Discussion

The prevalence of *TP53* mutations in cancer is well established, particularly in triple-negative breast cancer (TNBC), with certain mutation types consistently associated with poorer clinical outcomes across multiple cancers. Recent research highlights that p53 plays a non-transcriptional role regulating apoptosis by modulating intracellular Ca^2+^ dynamics at the ER and mitochondria [18,26]. Dysregulation of this Ca^2+^-dependent mechanism has been linked to apoptotic resistance, a hallmark of cancer progression [27]. Despite the prevalence of TP53 mutations, their differential impact on p53-mediated Ca^2+^ signalling remains understudied. This study addresses this gap by investigating the effect of various *TP53* mutations on Ca^2+^ channel gene expression, SOC, and their collective influence on TNBC apoptosis resistance. Our findings reveal that different *TP53* mutations alter Ca^2+^ homeostasis, highlighting a critical link between *TP53* status, SOCE dysregulation, and cancer progression.

Initial bioinformatic analysis identified a significant downregulation of *CACNA1D* in mutant *TP53* samples, particularly in TNBC, which was the only Ca^2+^ channel gene consistently altered across both patient and cell line datasets. We also noted that reduced *CACNA1D* expression was associated with poorer disease-free survival in BC, a trend that was observed in TNBC but failed to reach significance, likely due to the low N numbers in this subset. Thus, this work highlights the clinical target to be of potential importance in BC and TNBC. To date, only one other study has examined the impact of *TP53* status on ion channel expression in cancer [42]. Although this study was not TNBC focused, its findings align with ours, reporting downregulated *CACNA1D* expression in all BC *TP53* mutant samples [42]. This provides further evidence that this effect is not just TNBC-specific but wider BC samples; however, this association between mutant TP53 and *CACNA1D* has not been observed in other cancer types, requiring further work.

It is well documented compared to WT that different TP53 mutant types are associated with varying negative impacts on patient outcomes and phenotypes in cancer, and specifically TNBC [39,40]. Consequently, we sought to investigate the impact of this on *CACNA1D* expression using a panel of TNBC cells harbouring various *TP53* mutation types. We observed an upregulation of *CACNA1D* in cells harbouring missense mutations, but a downregulation in those with deleterious frameshift and stop mutations (Figure 1D). Intriguingly, *CACNA1D* mRNA expression also correlated with p53 protein levels, suggesting a relationship between both (Figure 1E). To our knowledge, this study is the first in cancer to propose a potential link between *CACNA1D* expression and p53, which is impacted by different *TP53* mutations. To validate this, further work is needed to employ larger BC datasets to discern how specific TP53 mutations impact *CACNA1D* expression and clinical outcomes in TNBC patient samples.

Due to the altered Ca^2+^ channel expression in *TP53* mutant TNBC cells, we sought to assess SOC in these models. Cell lines with stop and frameshift mutations showed reduced basal Ca^2+^ levels, ER store release, and SOCE, correlating with lower *CACNA1D* and p53 expression (Figure 1D,E and Figure 2A). In contrast, MDA-MB-468, harbouring a missense mutation, exhibited reduced basal Ca^2+^ and ER store release but increased SOCE, aligning with higher *CACNA1D* and *TP53* expression. TNBC cells with stop and frameshift *TP53* mutations had reduced proliferation rates and apoptotic sensitivity, while MDA-MB-468, which has a *TP53* SNV displayed increased levels of both (Figure 2E,F).

To confirm *TP53*’s role in regulating SOCE through *CACNA1D*, we treated *TP53* frameshift and stop mutated cell lines, which exhibited reduced p53 expression, with the p53 reactivator COTI-2. COTI-2 is a third-generation thiosemicarbazone that has been found to restore p53 protein expression and activity [36,43]. Upregulated *CACNA1D* expression in HDPQ-1 and MDA-MB-157 cells was associated with increased basal Ca^2+^ and SOCE, in MDA-MB-157 (Figure 3). This result aligns with previous studies demonstrating that p53 influences Ca^2+^ channel expression, particularly *TRPC6* and *TRPM4*, which also impacted intracellular Ca^2+^ levels. Further validation using *CACNA1D* knockdown in *TP53* WT CAL-51 cells (Figure 3E) significantly reduced SOCE, confirming its role in SOCE regulation. While no prior studies have demonstrated that *TP53* modulates *CACNA1D* expression to regulate SOCE, earlier research has shown that *CACNA1D* can influence SOCE in colon and prostate cancers, despite its conventional role as a voltage-gated Ca^2+^ channel [37,44].

Furthermore, there are few studies examining the effects of *TP53* mutations more generally on SOC in cancer. Giorgi et al. (2015) demonstrated in mouse embryonic fibroblasts that p53 knockout reduced mitochondrial Ca^2+^ levels, leading to apoptotic resistance to ER stressors [27]. Conversely, a study in colon cancer HCT-116 cells found no difference in SOC profiles between *TP53* WT and the missense mutation R175H [45]. However, they noted in their model that *TP53* overexpression significantly increased SOCE. Together, these findings, along with our study, highlight that p53 impacts SOC and that *CACNA1D* and *TP53* expression are closely linked to SOCE, but the effect varies depending on the mutation type. These findings underscore the need for further research using controlled models to better delineate the effects of specific *TP53* mutations on intracellular Ca^2+^ dynamics.

**Figure 5:**
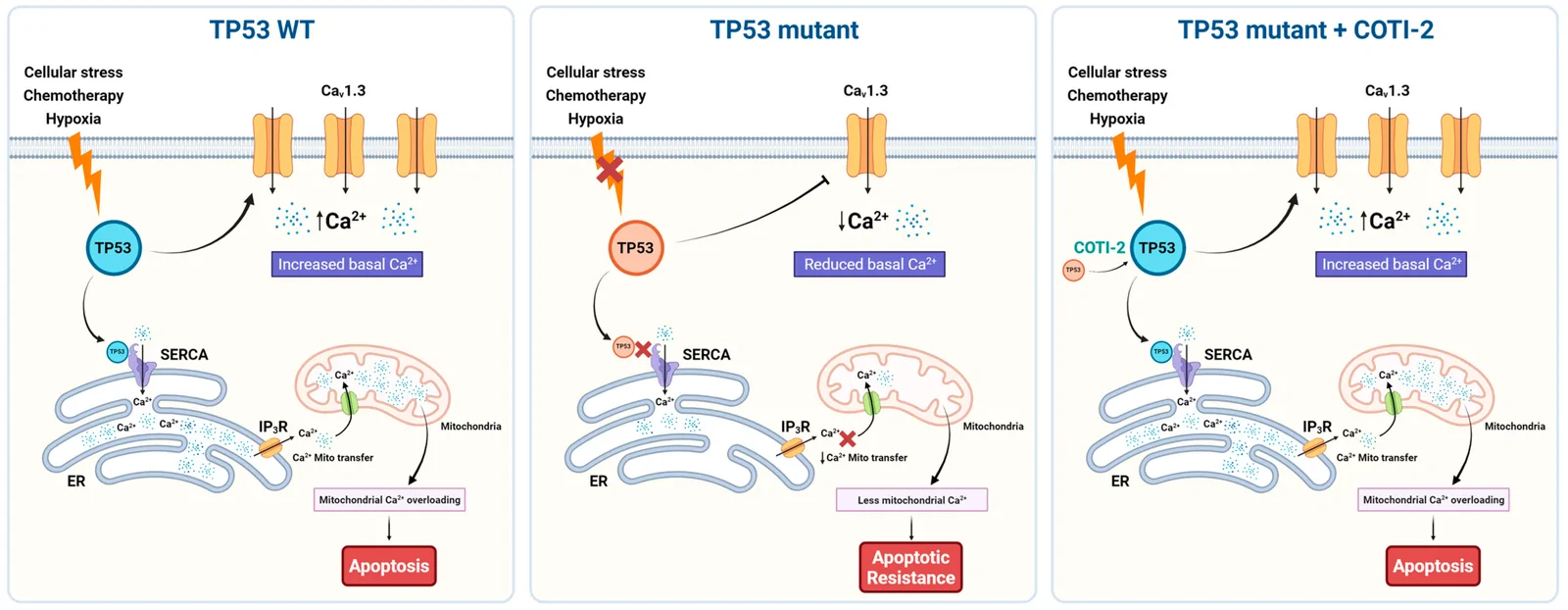
Schematic representation of TP53 mutation-dependent regulation of Ca^2+^ homeostasis and apoptosis via *CACNA1D*/Cav1.3 modulation in TNBC. Left (TP53 WT): In wild-type TP53 cells, cellular stress (e.g., chemotherapy, hypoxia) activates p53, which upregulates CACNA1D (Ca_V_1.3), leading to increased basal Ca^2+^ levels. Functional SERCA pumps and IP_3_Rs ensure proper ER Ca^2+^ handling and mitochondrial Ca^2+^ transfer, resulting in mitochondrial Ca^2+^ overload and apoptosis. Middle (TP53 mutant): In TP53 mutant cells (frameshift/stop), impaired p53 function leads to downregulation of Cav1.3 and SERCA activity, reducing basal and ER Ca^2+^ levels and limiting mitochondrial Ca^2+^ uptake. This dampens Ca^2+^-dependent apoptotic signaling, promoting apoptotic resistance. Right (TP53 mutant + COTI-2): Treatment with the p53 reactivator COTI-2 restores p53 activity in TP53 mutant cells, increasing Cav1.3 expression and basal Ca^2+^ levels. Enhanced Ca^2+^ transfer to mitochondria reinstates mitochondrial Ca^2+^ overload and apoptotic signaling, reversing apoptosis resistance.

Previous studies have demonstrated that reduced intracellular Ca^2+^ levels correlate with diminished apoptotic capacity [46,47], and *TP53* mutations are generally associated with chemotherapy resistance [48,49]. Our study aimed to investigate the impact of the *TP53*/Ca^2+^ axis on apoptosis resistance and whether restoring p53 function could reverse these effects. Focusing on *TP53* FS mutant cells, which exhibited significantly reduced *TP53*, *CACNA1D*, and SOC activity, we compared their response to *TP53* WT cells. Notably, treatment with a p53 reactivator significantly enhanced apoptosis in both *TP53* mutant and WT TNBC cells (Figure 4). Importantly, the combination of COTI-2 and TG elicited a synergistic apoptotic response across both cell lines, particularly in the MDA-MB-157 TP53 mutant cell line. This demonstrates that impaired apoptotic signalling in *TP53* FS mutant cells could be restored via *TP53* reactivation, which enhanced both *CACNA1D* expression and SOCE (Figure 5). Highlight targeting the Ca^2+^ machinery could be beneficial in restoring apoptosis capacity and enhancing responses to existing treatments in *TP53* mutant TNBC.

Our findings align with previous research demonstrating that COTI-2 exerts anti-proliferative effects in head and neck squamous cell carcinoma, ovarian and BC cell lines, including TNBC, both in vitro and in vivo [34,43,50,51]. Specifically, Synott et al. (2019) reported that COTI-2 preferentially induces apoptosis in TNBC cell lines, with lower IC_50_ values in p53 mutant cells compared to WT, mirroring our IC_50_ results (Figure S4B) [36]. While prior studies have highlighted COTI-2’s role in inducing apoptosis, to our knowledge, this is the first study to link its effects to enhanced *CACNA1D*-mediated SOC activity. While COTI-2 has demonstrated greater potency than other p53 reactivators such as APR-246, and superior specificity compared to broader compounds such as α-mangostin [35,36], further studies using a panel of these agents are needed to determine if they differentially affect SOC activity.

## 5. Conclusions

This study identifies a novel link between *TP53* mutations, Ca^2+^ channel expression, and altered SOCE in TNBC. We demonstrated that *TP53* mutation type significantly impacts Ca^2+^ signalling, with frameshift and stop mutations resulting in reduced expression of *CACNA1D* (encoding CaV1.3) and diminished SOCE. Restoration of *TP53* function using COTI-2 not only reinstated CaV1.3 expression and SOCE but also sensitised *TP53* mutant TNBC cells to apoptosis, highlighting the therapeutic potential of targeting dysregulated Ca^2+^ signalling pathways.

These findings underscore the importance of Ca^2+^ signalling in the context of *TP53* mutations, particularly the differential effects of distinct mutation types. Further studies in controlled TNBC models could help clarify the mechanistic links between *TP53* mutation subtypes, Ca^2+^ signalling, and therapeutic responses, while validating the combined use of *TP53* reactivators and Ca^2+^ modulators as a potential treatment strategy.

## Data Availability

The data presented in this study are available on request from the corresponding author.

## Supplementary Materials

The following supporting information can be downloaded at: https://www.mdpi.com/article/10.3390/cancers17101614/s1. Table S1: Primer sequences used to determine gene expression using RT-qPCR. Figure S1: Impact of low or high CACNA1D expression on disease free and overall survival in BC. Figure S2: Effect of COTI-2 treatment on calcium channel expression and SOCE in missense TP53 mutant TNBC cell lines. Figure S3: CACNA1D expression after siRNA knockdown. Figure S4: IC50 analysis for TG and COTI-2 in CAL-51 and MDA-MB-157 cells.

## Abbreviations

AC	All Cancer
BC	Breast cancer
CCLE	Cancer Cell Line Encyclopaedia
DEA	Differential expression analyses
DEG	Differentially expressed genes
DGEA	Differential Gene Expression Analysis
ER	Estrogen receptor
FBS	Fetal Bovine Serum
FS	Frameshift
HER2	Human epidermal growth factor receptor 2
LFC	Log2 fold change
Mut	Mutant
PM	Plasma Membrane
PR	Progesterone receptor
SERCA	Sarcoendoplasmic Reticulum Calcium ATPase pump
SNV	Single Nucleotide Variant
SOC	Store operated current
SOCE	Store operated calcium entry
TCGA	The Cancer Genome Atlas
TG	Thapsigargin
TNBC	Triple negative breast cancer
*TP53*	Tumour protein 53
TRP	Transient receptor potential channel
TSG	Tumour suppressor gene
WT	Wild-type
