# Correction: Eustace et al. TP53 Mutation-Specific Dysregulation of Store-Operated Calcium Entry and Apoptotic Sensitivity in Triple-Negative Breast Cancer. *Cancers* 2025, *17*, 1614

**DOI:** 10.3390/cancers18213558

**Published:** 2026-11-05

**Authors:** Kaneez E. Rabab, Paul J. Buchanan, Grace Colley, Anita White, Aisling Murphy, Chloe McCormack, Alex J. Eustace

**Affiliations:** 1 Life Sciences Institute, Dublin City University, D09 NR58 Dublin, Ireland; kaneez.rabab@gmail.com (K.E.R.); grace.colley3@mail.dcu.ie (G.C.); anita.white@dcu.ie (A.W.); aisling.murphy376@mail.dcu.ie (A.M.); chloe.mccormack44@mail.dcu.ie (C.M.); 2 School of Nursing, Psychotherapy and Community Health, Dublin City University, D09 Y8VX Dublin, Ireland; 3 School of Biotechnology, Dublin City University, D09 K20V Dublin, Ireland

## Supplementary Data Update

In the original published manuscript [1], Table S1 was inadvertently omitted from the Supplementary Materials. Table S1 has now been added to the Supplementary Materials. The authors state that the scientific conclusions are unaffected. This correction was approved by the Academic Editor. The original publication has also been updated.
