# Supplementary material for: TP53 Mutation-Specific Dysregulation of Store-Operated Calcium Entry and Apoptotic Sensitivity in Triple-Negative Breast Cancer
Source: Cancers (Basel). 2025 May 10;17(10):1614. doi: 10.3390/cancers17101614 (PMC12110487; doi:10.3390/cancers17101614)
Supplement: Supplementary file 1 [file cancers-17-01614-s001.zip › cancers-3577517-supplementary.pptx]

## Slide 1
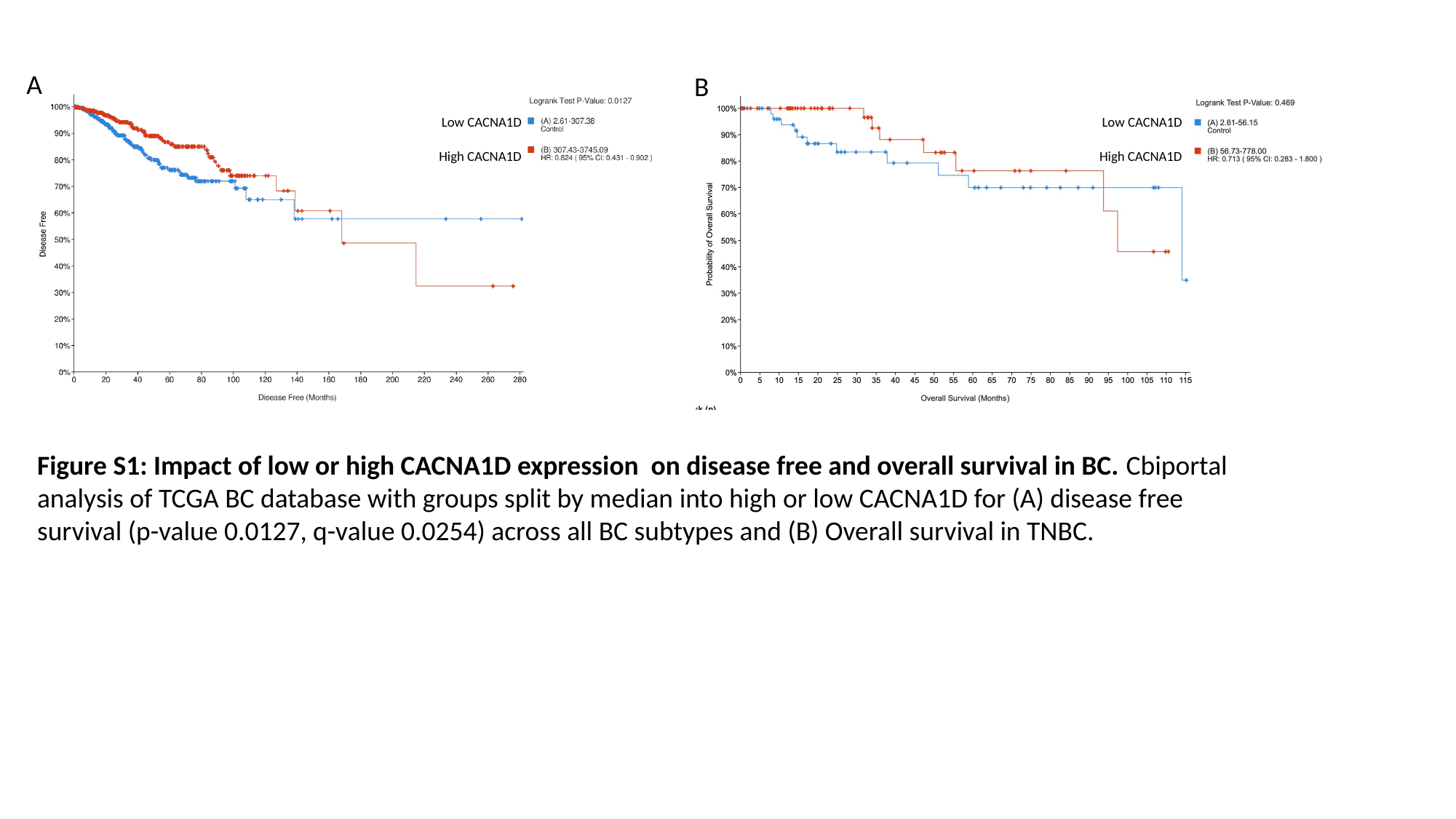

A
B
Low CACNA1D
Low CACNA1D
High CACNA1D
High CACNA1D
Figure S1: Impact of low or high CACNA1D expression on disease free and overall survival in BC. Cbiportal analysis of TCGA BC database with groups split by median into high or low CACNA1D for (A) disease free survival (p-value 0.0127, q-value 0.0254) across all BC subtypes and (B) Overall survival in TNBC.

## Slide 2
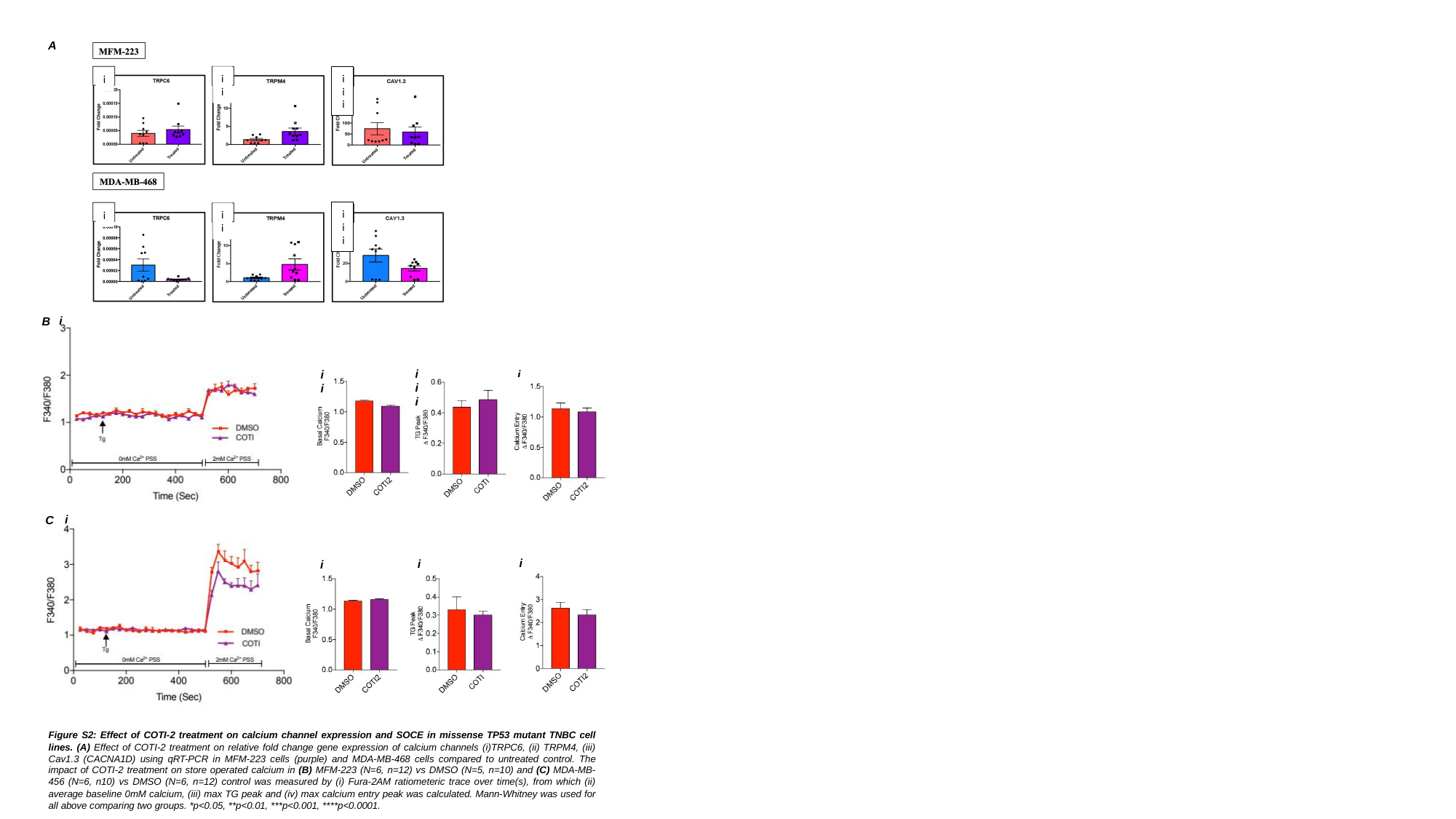

A
ii
iii
i
iii
ii
i
i
B
iii
iv
ii
i
C
iv
iii
ii
Figure S2: Effect of COTI-2 treatment on calcium channel expression and SOCE in missense TP53 mutant TNBC cell lines. (A) Effect of COTI-2 treatment on relative fold change gene expression of calcium channels (i)TRPC6, (ii) TRPM4, (iii) Cav1.3 (CACNA1D) using qRT-PCR in MFM-223 cells (purple) and MDA-MB-468 cells compared to untreated control. The impact of COTI-2 treatment on store operated calcium in (B) MFM-223 (N=6, n=12) vs DMSO (N=5, n=10) and (C) MDA-MB-456 (N=6, n10) vs DMSO (N=6, n=12) control was measured by (i) Fura-2AM ratiometeric trace over time(s), from which (ii) average baseline 0mM calcium, (iii) max TG peak and (iv) max calcium entry peak was calculated. Mann-Whitney was used for all above comparing two groups. *p<0.05, **p<0.01, ***p<0.001, ****p<0.0001.

## Slide 3
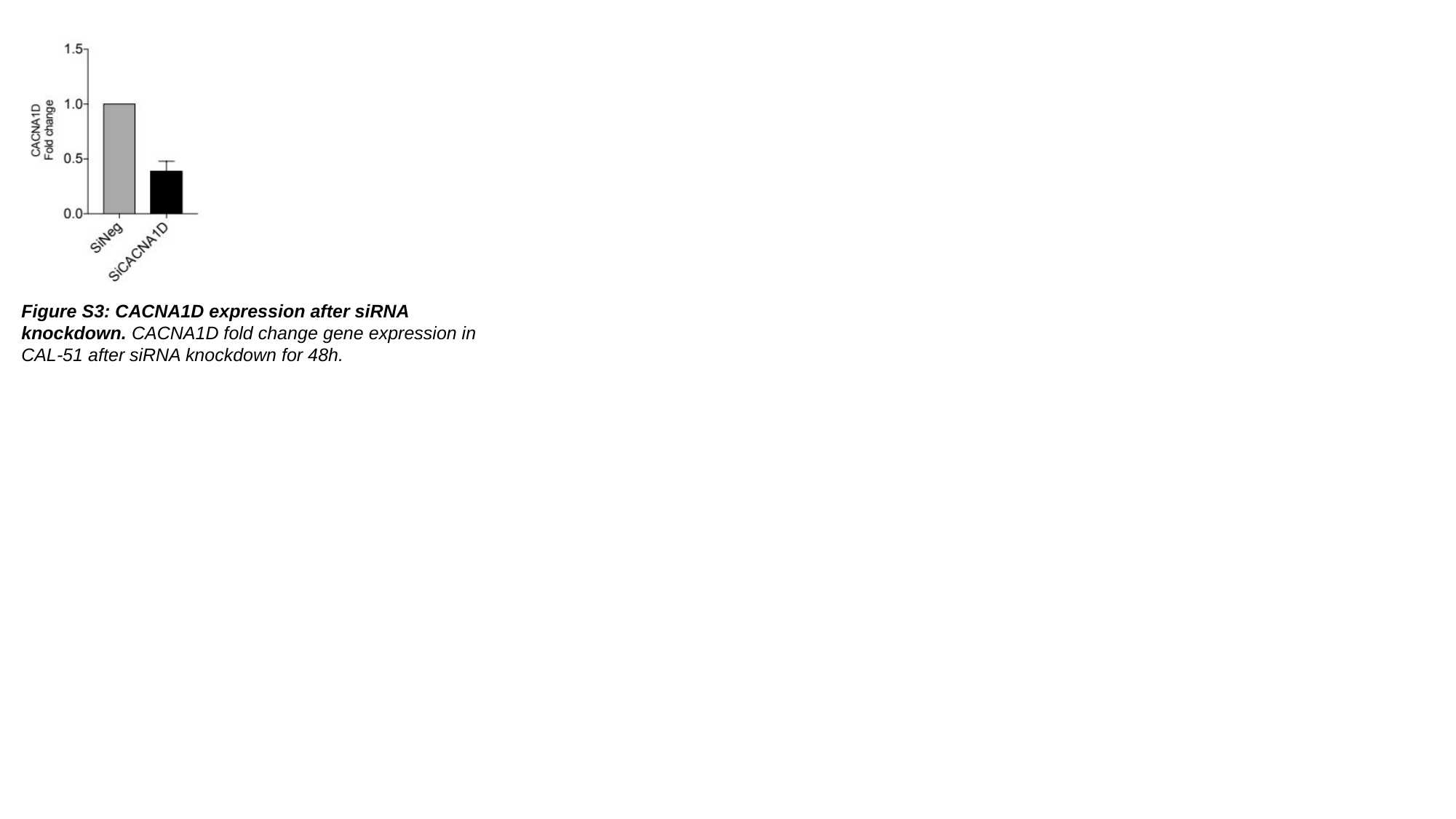

Figure S3: CACNA1D expression after siRNA knockdown. CACNA1D fold change gene expression in CAL-51 after siRNA knockdown for 48h.

## Slide 4
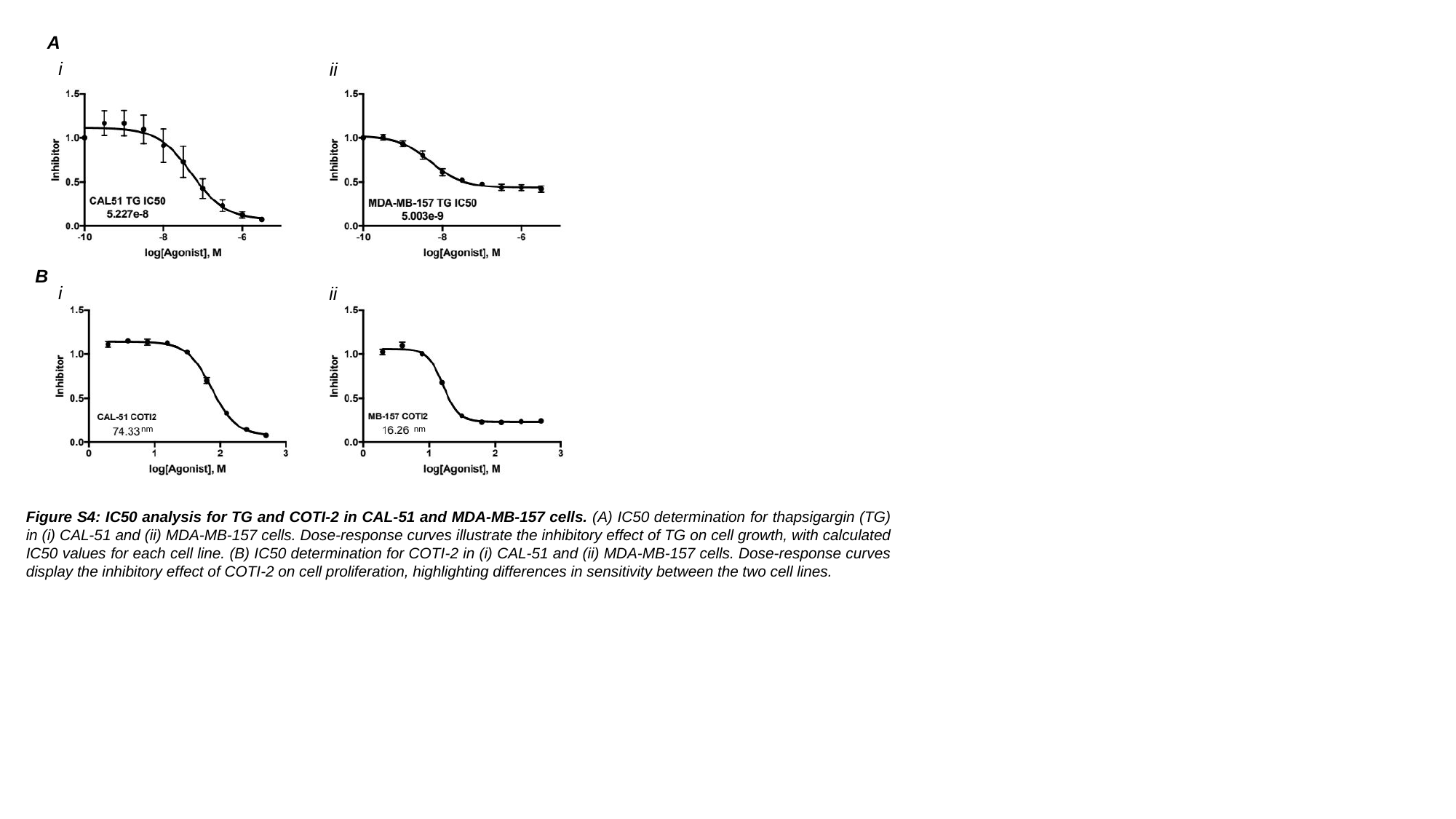

A
i
ii
B
i
ii
nm
nm
Figure S4: IC50 analysis for TG and COTI-2 in CAL-51 and MDA-MB-157 cells. (A) IC50 determination for thapsigargin (TG) in (i) CAL-51 and (ii) MDA-MB-157 cells. Dose-response curves illustrate the inhibitory effect of TG on cell growth, with calculated IC50 values for each cell line. (B) IC50 determination for COTI-2 in (i) CAL-51 and (ii) MDA-MB-157 cells. Dose-response curves display the inhibitory effect of COTI-2 on cell proliferation, highlighting differences in sensitivity between the two cell lines.
